# Juvenile Hormone and Insulin Regulate Trehalose Homeostasis in the Red Flour Beetle, *Tribolium castaneum*

**DOI:** 10.1371/journal.pgen.1003535

**Published:** 2013-06-06

**Authors:** Jingjing Xu, Zhentao Sheng, Subba Reddy Palli

**Affiliations:** Department of Entomology, College of Agriculture, University of Kentucky, Lexington, Kentucky, United States of America; University of California San Francisco, United States of America

## Abstract

Insulin/IGF-1 signaling (IIS) has been well studied for its role in the control of life span extension and resistance to a variety of stresses. The *Drosophila melanogaster* insulin-like receptor (InR) mutant showed extended life span due to reduced juvenile hormone (JH) levels. However, little is known about the mechanism of cross talk between IIS and JH in regulation of life span extension and resistance to starvation. In the current study, we investigated the role of IIS and JH signaling in regulation of resistance to starvation. Reduction in JH biosynthesis, JH action, or insulin-like peptide 2 (ILP2) syntheses by RNA interference (RNAi)-aided knockdown in the expression of genes coding for juvenile hormone acid methyltransferase (JHAMT), methoprene-tolerant (Met), or ILP2 respectively decreased lipid and carbohydrate metabolism and extended the survival of starved beetles. Interestingly, the extension of life span could be restored by injection of bovine insulin into JHAMT RNAi beetles but not by application of JH III to ILP2 RNAi beetles. These data suggest that JH controls starvation resistance by regulating synthesis of ILP2. More importantly, JH regulates trehalose homeostasis, including trehalose transport and metabolism, and controls utilization of stored nutrients in starved adults.

## Author Summary

Both juvenile hormone (JH) and Insulin/IGF-1 signaling (IIS) regulate life span and starvation resistance in insects. Regulation of longevity and starvation resistance by IIS has been well studied, yet little is known about the underlying mechanisms and cross talk between these two hormones. The red flour beetle, *Tribolium castaneum*, is a good model to study cross talk between JH and IIS because both of these pathways are important in regulation of life span and starvation resistance. The starved male beetles with either reduced JH or ILP2 levels live longer due to a lower rate in lipid and carbohydrate metabolism when compared with the control beetles. Juvenile hormone regulates starvation survival through regulation of synthesis of ILP2, trehalose transporter (TRET), and trehalase. Reduction in JH levels or its action or ILP2 expression decreased trehalase levels in the fat body, resulting in a slower rate of conversion of trehalose to glucose. Reduction in JH levels or its action also caused a decrease in TRET levels in the alimentary canal leading to a lower rate of uptake of trehalose into this tissue resulting in more trehalose available in the hemolymph. Trehalose likely regulates various processes to protect beetles from stress.

## Introduction

Many biological functions of juvenile hormone (JH) in regulation of almost every aspect of an insect's life have been reported since its discovery in 1965 [1], [2]. To maintain the larval state, JH induces the expression of the genes coding for transcription factors such as Kr-h1 to prevent metamorphosis; knockdown in the expression of the gene coding for Kr-h1 by RNAi in larvae leads to precocious metamorphosis that cannot be rescued by exogenous JH application [3]. During the larval stage, JH suppresses imaginal disc growth promoted by nutrition [4], and the nutritional signals mediated by insulin/IGF signaling (IIS) can override JH suppression [5]; but, in the absence of JH, the wing disc grows despite severe starvation. Interestingly, the wing disc growth is well correlated with trehalose levels during the larval stage until the critical weight is reached; starvation causes a decline in hemolymph glucose and trehalose and cessation of wing imaginal disk growth, which can be rescued by injection of trehalose. After reaching the critical weight, the trehalose response to starvation disappears and the action of insulin becomes decoupled from nutrition. The wing disks also lose their sensitivity to repression by JH [6].

To direct reproductive maturation in *Drosophila melanogaster* and *Tribolium castaneum*, JH regulates the production of male accessory gland proteins in the male and vitellogenin (Vg) in the female [7]–[9]. A basic helix-loop-helix (bHLH) per-Arnt-Sim (PAS) family transcription factor, methoprene-tolerant (Met) interacts with other members of this family including steroid receptor co-activator (SRC) and Cycle; binds to both JH and JH response elements (JHRE) present in the promoters of JH-response genes [10]–[15].

The function of JH has been well studied in the regulation of molting, metamorphosis, and reproduction. However, mechanisms of JH action in regulation of life span and starvation resistance are still unclear. In the monarch butterfly, migrant adults live longer than summer adults when both are maintained under standard laboratory conditions. Interestingly, the longevity of migrant adults is restored to that of summer adults by treatment with JH I, and the life span of summer adults is increased by 100% when the corpora allata are surgically removed [16]. Similarly, in the InR mutant of *D. melanogaster*, life span extension is due to reduced JH levels [17]. These studies showed that lower levels of JH could extend the life span under certain conditions. However, the underlying mechanisms of JH action on the longevity and the cross talk between JH and IIS pathway are still not well understood.

The IIS function in life span, longevity, and stress resistance has been thoroughly investigated because of evolutionarily conserved function from yeast to mammals [18]. These functions include regulation of cellular adaptation to stress stimuli, such as nutrient-poor conditions [19] and oxidative stress [20], [21], promoting autophagy [22] and regulation of metabolism [23].

*T. castaneum* is a good model for these studies because of efficient functioning of RNAi and rapid JH response. In the previous studies, we showed that JH regulates ILP2 and ILP3 synthesis; ILP2 and ILP3 in turn regulate Vg synthesis [9]. These studies provided a good model to explore the interplay between JH and IIS signaling pathways. Here, we investigated the effects of JH and IIS on survival and carbohydrate metabolism in adults under starvation. RNAi, topical application of JH III, and injection of bovine insulin were used to modify JH and/or insulin levels in the adults of *T. castaneum* to study the cross-talk between JH and IIS signaling in regulation of resistance to starvation.

## Results

### Juvenile hormone and insulin-like peptide promote survival of starved beetles

**Figure 1:**
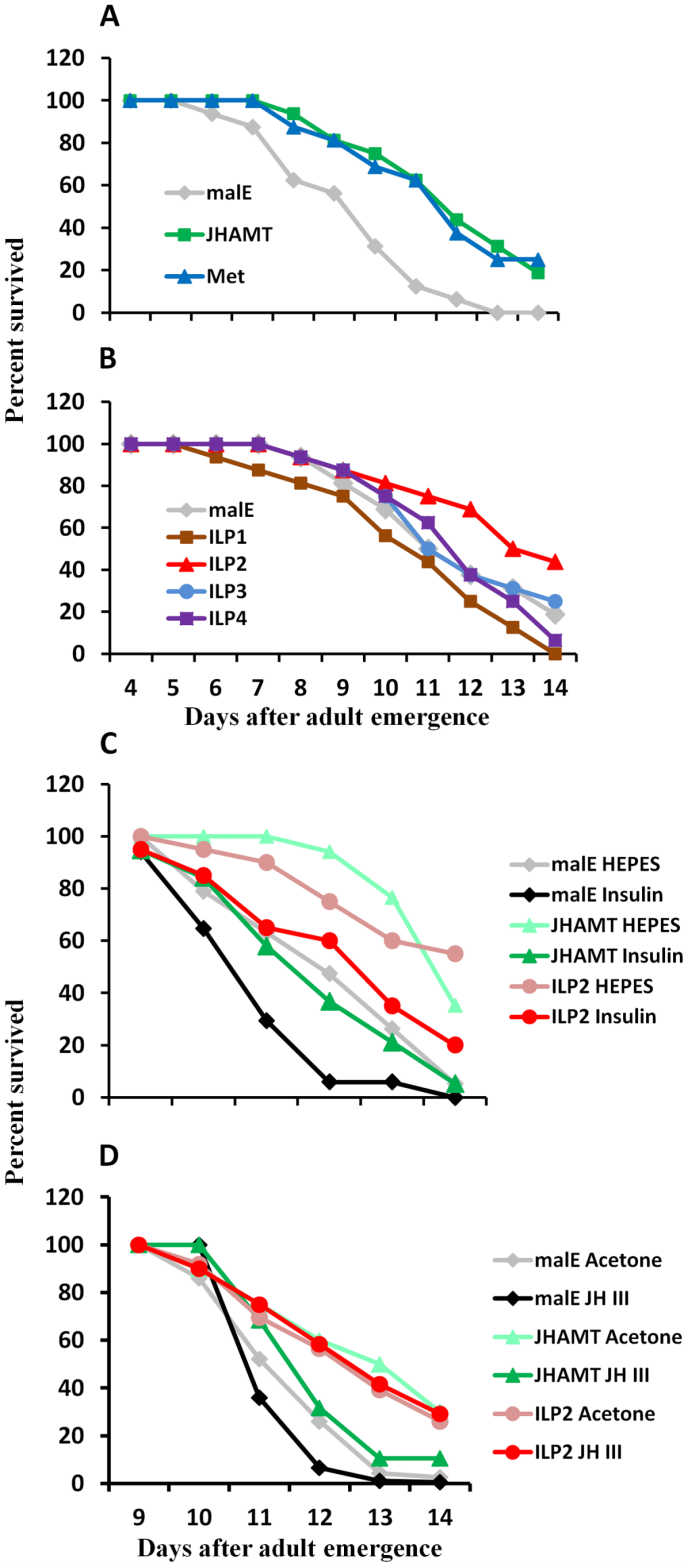
JH and insulin regulate starvation resistance. A. RNAi-aided knockdown in the expression of genes coding for JHAMT or Met extended the starvation survival. Percent beetles survived during starvation after injection of control *malE*, JHAMT, or Met dsRNA respectively into 40 newly emerged male adults are shown. Starvation survival was recorded from day 4 to day 14. B. RNAi-aided knockdown in the expression of genes coding for ILP2 extend starvation survival. Percent beetles survived during starvation after injection of control *malE*, ILP1, ILP2, ILP3, or ILP4 dsRNA respectively into 40 new emerged male adults are shown. Starvation survival was recorded from day 4 to day 14. C. Bovine insulin can rescue the starvation survival of JHAMT or ILP2 RNAi beetles. Percent beetles survived after injection of control *malE*, JHAMT, ILP2 dsRNA into day 0 male adults followed by injection of either 25 mM HEPES or 25 mM HEPES containing 10 mg/ml insulin into day 5 adults are shown. The starvation survival was recorded from day 9 to day 14. D. JH III rescues starvation survival in JHAMT but not in ILP2 RNAi beetles. Shown are percentages of beetles survived after injection of control *malE*, JHAMT, ILP2 dsRNA into day 0 male adults followed by topical application of either acetone or 10 mM JH III in acetone on days 3, 5, and 7. The starvation survival was recorded from day 9 to day 14.

To determine the role of JH in the survival of the starved *T. castaneum*, the newly emerged male adults were injected with *malE* (dsRNA prepared using a bacterial gene *malE* as a control), JHAMT (a key enzyme in JH synthesis), and Met (JH receptor). The control-starved beetles began to die on seventh day post-adult emergence (PAE), and all beetles died by the fourteenth day PAE. However, a block in JH synthesis or its action by knockdown in the expression of genes coding for JHAMT (mean survival 12.8 days) or Met (mean survival 12.7 days) extended survival of the starved beetles by one day (control mean survival 11.7 days, *P* = 0.00002 in log rank test, Fig. 1A).

To determine whether or not ILPs are involved in regulation of starvation survival, ILP1, ILP2, ILP3, and ILP4 dsRNA were injected into newly emerged adults, and the survival of RNAi beetles was monitored under starvation conditions. All four dsRNAs caused more than 80% reduction in their target mRNA levels (Fig. S1). As shown in Figure 1B, only ILP2 knockdown extended life span (mean survival 12.9 days) similar to that in JHAMT or Met RNAi beetles when compared with the control (12.1 days mean survival, *P* = 3.39E-06 in log rank test). While ILP1 knockdown shortened the survival, ILP3 and ILP4 knockdown did not show any significant effect. In addition, injection of bovine insulin decreased survival of JHAMT (12.9 days mean survival) and ILP2 RNAi beetles (12.6 days mean survival) to that in control insects (12.1 days mean survival, *P* = 0.005, Fig. 1C). The application of JH III decreased the survival of JHAMT RNAi beetles (from 11.6 to 11.0 days mean survival, *P* = 0.038), but not ILP2 RNAi beetles (11.5 days mean survival for both, *P* = 0.467). (Fig. 1D). These data suggest that both IIS and JH may work through similar or overlapping mechanisms to regulate survival of starved adults and that JH may work upstream to the IIS pathway.

### JH and IIS regulate metabolism in the starved beetles

**Figure 2:**
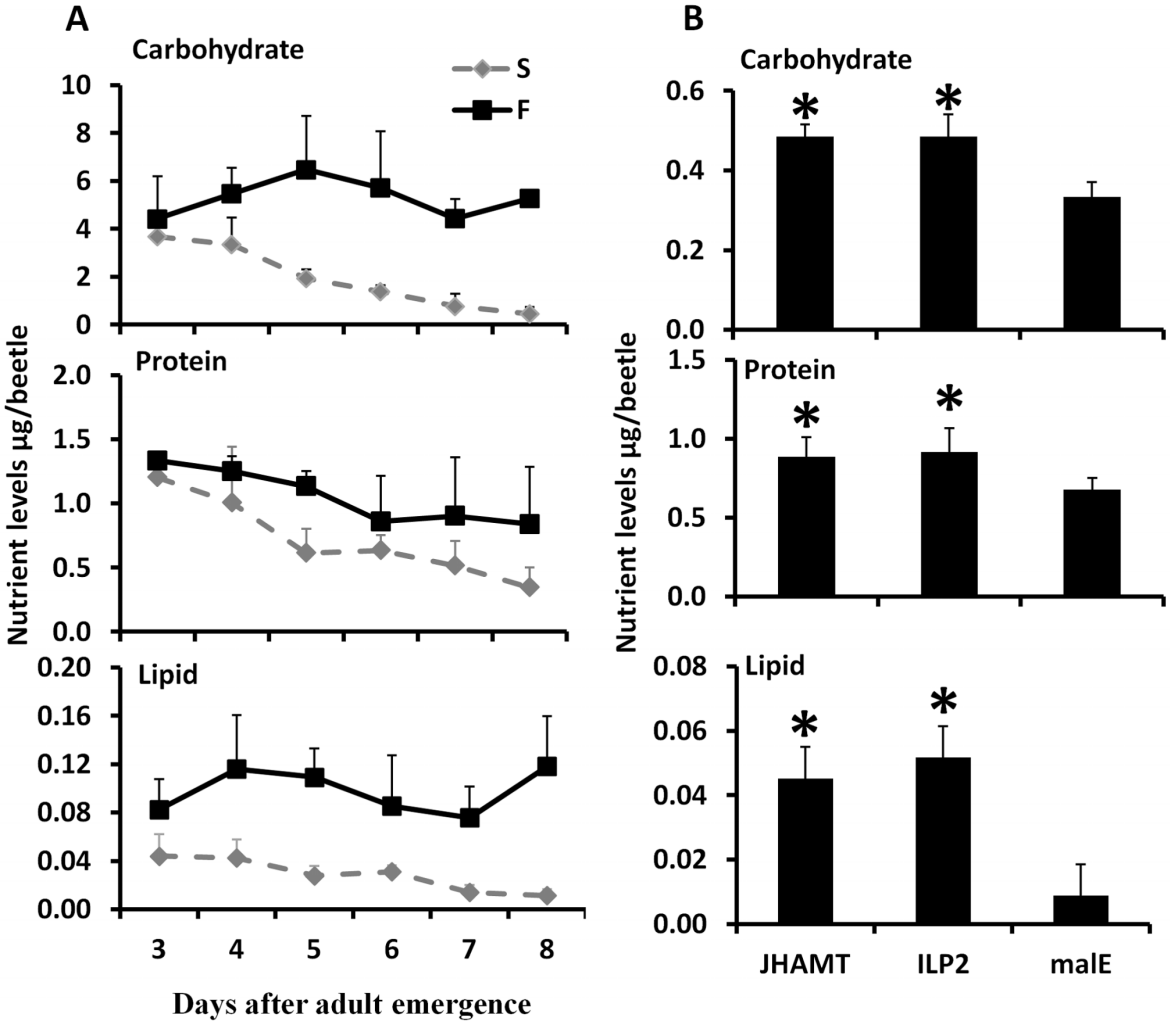
JH and ILP2 regulate carbohydrate, protein, and lipid metabolism during starvation. A. Total carbohydrate, protein, and lipid levels were determined by Anthrone reagent, Bradford, and vanillin reagent respectively in samples collected from day 3 to day 8 starved and fed male beetles. Three beetles were used for each time point and six biological replicate were used. The Means+S.D (n = 6) are shown. B. The nutrient levels including carbohydrate, protein, and lipid are regulated by JH and ILP2 during starvation. The male beetles injected with *malE*, ILP2, or JHAMT dsRNA were collected on day 8. The total carbohydrate, protein, and lipid were determined. Shown are the Means+S.D (n = 6). Asterisks show treatments that are significantly different (*P*<0.05) by one-way ANOVA.

To determine the major energy source for starved beetles, the total lipid, carbohydrate, and protein levels were measured in fed and starved beetles. In the fed beetles, the levels of all three nutrients did not change significantly during days 3–8 PAE (Fig. 2A). In contrast, in the starved animals, the levels of all three nutrients gradually decreased from day 3 to day 8 PAE (Fig. 2A). These data suggest that the beetles use all three sources of nutrients during starvation. To determine whether JH or IIS regulate metabolism of these macromolecules, the levels of these macromolecules were determined in JHAMT or ILP2 RNAi beetles. Strikingly, higher protein, carbohydrate, and lipid levels were detected in JHAMT and ILP2 RNAi beetles when compared to the levels in the control beetles injected with *malE* dsRNA (Fig. 2B). These data suggest that the life span extension during starvation in either JH or IIS deficient animals could be due to the reduced metabolism.

### Juvenile hormone regulates starvation survival via trehalose homeostasis

**Figure 3:**
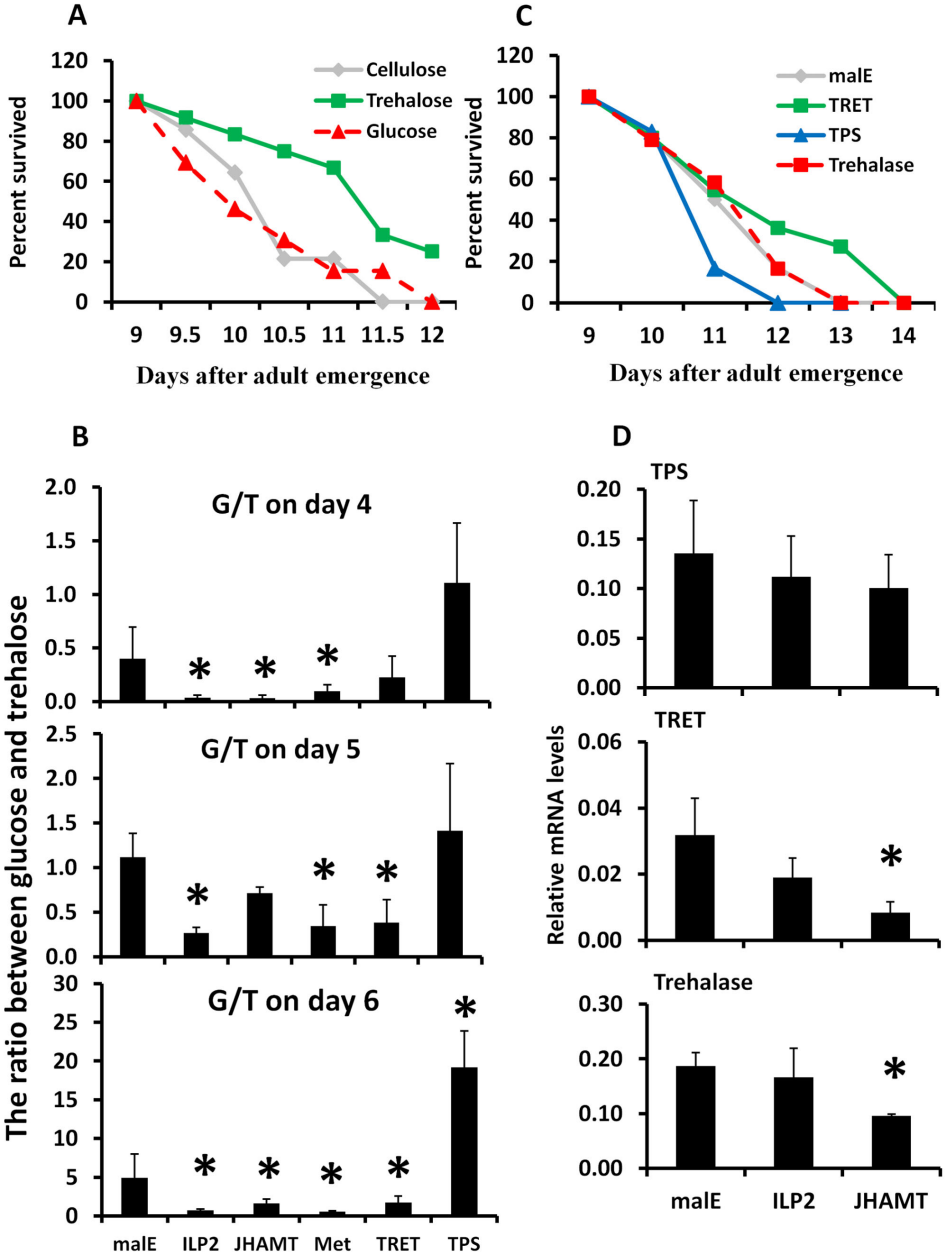
Trehalose metabolism plays an important role in extending life span during starvation. A. Starvation survival after feeding 10% trehalose plus cellulose, 10% glucose plus cellulose, or cellulose alone to the 8-day-old starved male beetles. The survival was recorded from day 9 to day 12. B. The ratio between hemolymph glucose and trehalose on days 4, 5, and 6 after injection of *malE*, ILP2, JHAMT, Met, TRET, or TPS dsRNA into the newly emerged adults. The hemolymph was extracted from three beetles for each treatment. The trehalose concentrations were determined using the glucose reagent and trehalase. The data shown are the Mean+S.D. (n = 6). C. Starvation survival after manipulation of endogenous trehalose level by injecting TRET, TPS, or trehalase dsRNA on day 0. The beetles were starved until 14 days. The survival was recorded from day 9 to day 14. D. The relative mRNA level of TPS, TRET, and trehalase after injecting *malE*, JHAMT, or ILP2 dsRNA. Total RNA was isolated on day 5 from beetles injected with control *malE*, JHAMT, or ILP2 dsRNA and starved. The RNA was converted to cDNA, and the relative levels of TPS, TRET, and trehalase mRNA were determined by qRT-PCR using RP49 as a control. The data shown are the Mean+S.D. (n = 3). Asterisks show treatments that are significantly different from the control (*P*<0.05) in one-way ANOVA.

To determine whether the trehalose, a major sugar in most insects, or the glucose, a major sugar in most animals, is utilized during starvation, trehalose or glucose were fed to the starved beetles. When beetles were fed on non-nutritional cellulose diet or cellulose diet supplemented with 10% trehalose or 10% glucose, the beetles fed on a trehalose-supplemented diet lived significantly longer when compared to the other two groups (*P* = 0.001, Fig. 3A). There was no significant difference in the survival of cellulose-fed or cellulose+10% glucose-fed beetles (Fig. 3A). These data suggest that major insect sugar trehalose is important for survival of starved beetles. Moreover, the ratio of glucose and trehalose in the hemolymph increased in the control beetles upon starvation from day 4 to day 6, suggesting more glucose is needed during starvation for the energy supply. However, this ratio decreased by 77–81%, 37–93%, and 70–89% in starved ILP2, JHAMT, or Met RNAi beetles respectively, when compared to the levels in control beetles (Fig. 3B). These data suggest that JH and ILP2 regulate trehalose levels in starved beetles.

Trehalose homeostasis is controlled by trehalose-6-phosphate synthase (TPS), the main enzyme involved in the synthesis of trehalose in the fat body [24]; Trehalose transporter (TRET), the direction of transport depends on the concentration gradient of trehalose [25]; and the trahalase, the major enzyme involved in conversion of trehalose to glucose in various insect tissues [26]. To determine the relative contribution of TRET, TPS, and trehalase in extending life span in starved beetles, we identified genes coding for trehalase (G04791), TRET (G13653), and TPS (G07883) based on sequence similarity with their homologs in other insects. These genes are highly conserved among insects (Fig. S2). We injected trehalase, TRET or TPS dsRNA into newly emerged beetles. The dsRNA injected beetles were starved for 13 days and life span changes were monitored. The TRET RNAi beetles showed a slight but significant increase by 0.26 day of mean survival in life span when compared to the control beetles (*P* = 0.042, Fig. 3C). Trehalase RNAi beetles showed no differences from the control, and the TPS RNAi beetles showed a decrease by 0.21 day of mean survival in life span when compared to the control beetles (*P* = 0.05, Fig. 3C). Similarly, the ratio between glucose and trehalose decreased by 44–66% in TRET RNAi beetles and increased by 1.2–3.9-fold in TPS RNAi beetles during starvation (Fig. 3B). RNAi studies showed that the mRNA levels of TRET and trehalase, but not TPS, decreased in beetles injected with JHAMT dsRNA, suggesting that JH is required for expression of genes coding for TRET and trehalase during starvation (Fig. 3D).

**Figure 4:**
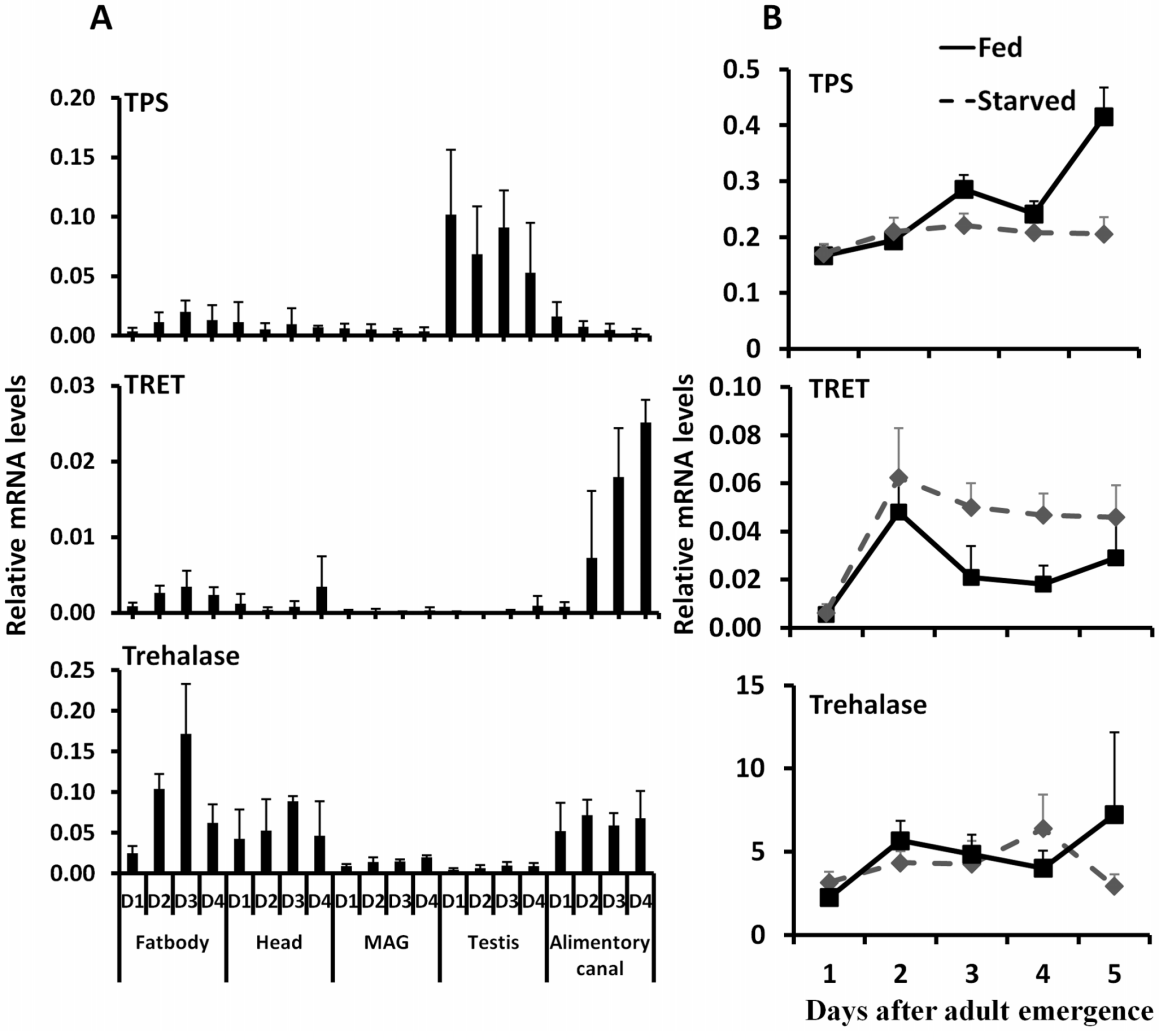
The relative mRNA levels of TRET, TPS, and trehalase in the fat body, head, male accessory gland, testis, and alimentary canal. A. Total RNA was isolated from the tissues dissected from the fed beetles from day 1 to day 4, the total RNA was converted to cDNA, and the relative TPS, TRET, and trehalase mRNA levels were determined by qRT-PCR using RP49 as a control. The data shown are the Mean+S.D. (n = 3). Asterisks show treatments that are significantly different from the control (*P*<0.05) in one-way ANOVA. B. The relative mRNA level of TRET, TPS, and trehalase in the starved and fed male beetles collected on day 1 to day 5 after adult emergence. Total RNA was isolated from the whole body of both fed and starved beetles on days 1 to 5. The total RNA was converted to cDNA, and the relative TPS, TRET, and trehalase mRNA levels were determined by qRT-PCR using RP49 as a control. The data shown are the Mean+S.D. (n = 3).

Studies on expression of genes coding for TPS, TRET, and trehalase in male adults showed that gene coding for TPS is predominantly expressed in the testis, gene coding for TRET is predominantly expressed in the alimentary canal, and gene coding for trehalase is expressed in the fat body, head, and alimentary canal (Fig. 4A). Comparison of TPS, TRET, and trehalase mRNA levels in starved and fed adults showed that TRET mRNA levels are higher in the starved beetles when compared to their levels in fed beetles. In contrast, the TPS mRNA levels are higher in the fed beetles than in the starved beetles. However, trehalase mRNA levels did not vary between starved and fed beetles (Fig. 4B).

**Figure 5:**
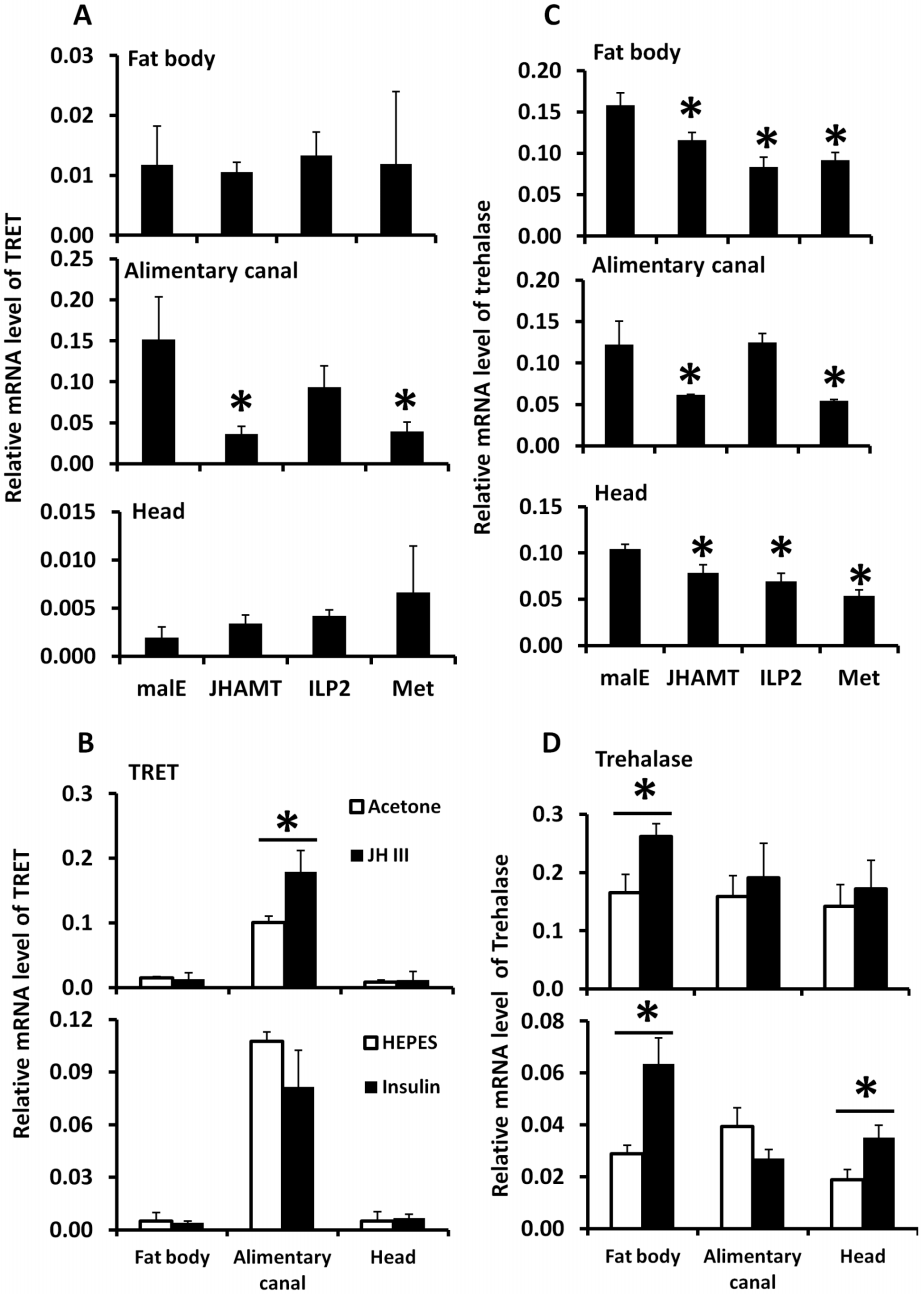
The relative mRNA levels of TRET and trehalase in the alimentary canal, fat body, and head after manipulation of JH or insulin levels. A. The relative mRNA levels of TRET in the alimentary canal, fat body, and head after injection of *malE*, JHAMT, ILP2, or Met dsRNA into newly emerged male adults. Total RNA was isolated and used to measure relative TRET mRNA by qRT-PCR using RP49 as a control. The data shown are the Mean+S.D. (n = 3). B. The relative mRNA levels of TRET in the alimentary canal, fat body, and head after topical application of 0.5 µl acetone, 0.5 µl 10 mM JH III in acetone, or injection of 0.3 µl HEPES solution or bovine insulin in HEPES solution at 10 mg/ml concentration. Total RNA was isolated from the tissues including fat body, alimentary canal, and head dissected from the starved beetles at 6 hours after treatment on day 5 for JH and insulin induction experiment. The total RNA was converted to cDNA, and the relative TRET mRNA levels were determined by qRT-PCR using RP49 as a control. The data shown are the Mean+S.D. (n = 3). C. Same treatments as in Figure 5A except trehalose mRNA levels were determined. D. Same treatments as in Figure 5B except trehalose mRNA levels were determined.

TRET mRNA levels decreased in JHAMT and Met RNAi insects when compared to their levels in control insects in the alimentary canal but not in the fat body or head of starved insects, suggesting that JH regulates the expression of this gene in the alimentary canal (Fig. 5A). Moreover, topical application of JH III induced the expression of the gene coding for TRET in the alimentary canal but not in the fat body or head (Fig. 5B). Similarly, the mRNA levels of trehalase in the fat body decreased in JHAMT, Met, and ILP2 RNAi insects (Fig. 5C). A decrease in mRNA levels of trehalase was observed in the alimentary canal isolated from JHAMT and Met RNAi beetles (Fig. 5C). Similarly, head tissue dissected from JHAMT, ILP2, and Met RNAi insects showed a decrease in trehalase mRNA levels (Fig. 5C). Topical application of JH III induced the gene coding for trehalase in the fat body but not in the alimentary canal or head (Fig. 5D). Injection of insulin into starved males on day 5 induced trehalase gene expression by 2.2 and 1.9-fold in the fat body and head respectively when compared to the levels in the same tissues dissected from control beetles (Fig. 5D). These data suggest that both JH and insulin regulate expression of the gene coding for trehalase and JH but not insulin regulates expression of the gene coding for TRET.

## Discussion

### JH and ILP2 regulate trehalose homeostasis

The first major contribution of the current study is the discovery that JH and ILP2 regulate trehalose homeostasis in starved beetles. RNAi-aided knockdown in the expression of genes coding for JHAMT (a key enzyme in JH synthesis) and Met (JH receptor) or ILP2 extended the survival of starved beetles (Fig. 1&S4). Injection of bovine insulin rescued the effects of both JHAMT and ILP2 RNAi on starvation survival. In contrast, topical application of JH III restored starvation resistance to the control level in starved JHAMT RNAi adults, but not in the ILP2 RNAi adults (Fig. 1C & D). RNAi-aided knockdown in the expression of genes coding for JHAMT or Met in male adult beetles caused a decrease in expression of ILP2 suggesting that both JH and its receptor are required for expression of this gene (Fig. S3). In addition, topical application of JH III induces expression of ILP2 in male beetles (Fig. S3). Moreover, JH titer could have been higher in starved beetles than the titers in the fed beetles as suggested by both JHAMT and Kr-h1 mRNA levels (Fig. S5). Taken together, these data suggest that JH regulates starvation resistance at least partially working through ILP2. Similar results on the role of JH in starvation resistance and extending life span have been reported in the burying beetles including *Nicrophorus orbicollis*, *N. tomentosus*, and *Ptomascopus morio* [27]; in *D. melanogaster* [17], [28]; and in the monarch butterfly [16]. In both *T. castaneum* [9] and *Apis mellifera* [29], JH induces expression of ILPs. In *T. castaneum* JH induces expression of ILP2 and ILP3 in females and regulates expression of Vg genes through insulin pathway. In *A. mellifera*, JH works through ILP1 and regulates carbohydrate metabolism when worker bees shift from nursing to foraging. The conserved roles of IIS pathway have been well studied in regulation of life span and reproduction from yeast to mammals [18], [29], [30]. In *D. melanogaster*, partial ablation of the insulin producing cells, the median neurosecretory cells in the brain, has extended life span, reduced fecundity, altered lipid and carbohydrate metabolism and increased oxidative stress resistance [31], [32]. These previous studies and our data reported in this paper suggest that JH regulates metabolic and reproductive processes at least partially through IIS signaling pathway.

How does JH regulate carbohydrate metabolism? In *T. castaneum* males, JH regulates expression of genes coding for trehalase and TRET, the two proteins critical for trehalose metabolism and transport (Fig. 5). Knockdown in expression of genes coding for JHAMT, Met, or ILP2 in the starved *T. castaneum* caused a decrease in trehalase mRNA levels in the fat body, which is the major tissue for storage of nutrients (Figs. 3 & 5). This would have caused a decrease in metabolism of trehalose to glucose, resulting in an increase in trehalose and decrease in glucose in the hemolymph. In *T. castaneum*, JH regulates expression of the gene coding for trehalase through the ILP2 and IIS pathway.

Interestingly, JH but not ILP2 regulates expression of the gene coding for TRET in *T. castaneum*, suggesting that JH may recruit Met to bind the promoter region of the gene coding for TRET, which is a different mechanism from that described for trehalase regulation (Fig. 5). Studies are in progress to test this hypothesis. Our data suggest that IIS is not involved in transcriptional regulation of the gene coding for TRET. However, it is possible that IIS may regulate translocation of TRET protein similar to insulin regulation of glucose transporter 4 (GLUT4) in humans by stimulating translocation of GLUT4 to the plasma membrane [33]–[35]. In type II diabetes patients, expression levels of the gene coding for GLUT4 and its translocation influence glucose transport [36], [37].

Insulin regulation of trehalose levels has been reported in *Caenorhabditis elegans*, *D. melanogaster*, and *Bombyx mori* [31], [38]–[41]. Insulin signaling regulates trehalose homeostasis by controlling expression of the gene coding for trehalase in the silkworm *B. mori* [42], [43] by a direct molecular interaction with trehalase in *Tenebrio molitor* [26] and by regulating the trehalose synthesis in *C. elegans* [39]. However, in the starved male *T. castaneum*, knockdown in the expression of the gene coding for either ILP2 or JHAMT did not affect TPS mRNA levels, suggesting that TPS is not under the control of JH or IIS in starved male beetles. It is possible that trehalose synthesis, an energy consuming process, is not active during starvation.

### Trehalose functions in the starvation resistance

The second major contribution of the current studies is the discovery that trehalose plays an important role in starvation resistance in *T. castaneum*. Feeding trehalose but not glucose extended the starvation survival, suggesting that trehalose plays an important role in starvation resistance in addition to being an energy source. Trehalose alters the life span as shown in both IIS-reduced *C. elegans* and JH-deficient fruit fly [38], [39]. In addition to the main function as an energy source [24], [44], trehalose could be acting as a chemical chaperone or as a metabolism modifier in protection of beetles from death.

Insect hemolymph as a “sink” or “reserve” carries a variety of metabolites [45]–[47]. The hemolymph composition of metabolites reflects nutrient intake and serves as a feedback signal to regulate food intake [48] and the rate of energy expenditure [49]. It is also possible that the trehalose distributed to the tissues and organs could help protect cells against heat, cold, desiccation, anoxia, and oxidation and retard age-associated decline in survivorship and extend life span [50]. In addition, trehalose induces autophagy, independent of TOR, clears the aggregate-prone proteins associated with Parkinson's [51] and Huntington's [52] diseases.

The trehalose homeostasis including trehalose levels and trehalose distribution play important roles in starvation resistance. Here, we found that both IIS and JH signaling pathways are involved in controlling starvation resistance via regulating trehalose homeostasis. The detailed molecular mechanisms that govern the cross-talk between these two major signaling pathways in regulation of trehalose homeostasis are the focus of intense research in several laboratories around the world.

## Materials and Methods

### *Tribolium castaneum* rearing and staging

Strain GA-1 of *T. castaneum* was reared on organic wheat flour containing 10% yeast at 30±1°C under standard conditions. New adults were separated within 6 hours post-adult eclosion (PAE) and staged from then onward.

### RNA isolation, cDNA synthesis, quantitative reverse transcriptase PCR and double-stranded RNA synthesis

Total RNA was isolated using the TRI reagent (Molecular Research Center Inc., Cincinnati, Ohio). The DNA was eliminated from the total RNA using DNase I (Ambion Inc., Austin, Texas) and 2 µg of total RNA for each sample was used for cDNA synthesis. Primers used in quantitative reverse transcriptase PCR (qRT-PCR) are listed in Table 1 or previously published [9], [12]. QRT-PCR reactions were performed using a common program as follows: initial incubation of 95°C for 3 min was followed by 40 cycles of 95°C for 10 s, 55°C for 1 min, Relative levels of mRNAs were quantified in triplicates and normalized using an internal control (ribosomal protein 49, RP49 mRNA).

**Table 1:** Primers used for dsRNA and real time PCR.

Genes		Primer sequence(5′-3′)
TRET-qRT-PCR	F	TAATCGATCGTTGTGGGAGACGCT
(G13653)	R	TAGGCTGCAGTATTTCGCCCAAGT
TRET-dsRNA*	F	GGAGCCGTCAACTTTGCATCAACA
(G13653)	R	TCTCCAACCACGTCCTTGAACAGT
trehalase-qRT-PCR	F	GCGCTCCAACTACAAAGCGTTCAA
(G04791)	R	TCCATGATGATCCCGTTCGTCCAA
trehalase-dsRNA*	F	ACCCGACTAATATTGCGCCACTGT
(G04791)	R	CGTGTTGTTCAGGCCCACAATCAT
TPS-qRT-PCR	F	GCAACTTTGACAATGTCGTCGCCT
(G07883)	R	ACCCACCCATAAGCCATTACCGTT
TPS-dsRNA*	F	AAGGCGTTGAAGTCTCTCCCGAAA
(G07883)	R	TTTACGGTCGACACGACATCCCAA

* The T7 promoter sequence was added at the 5′ end of each dsRNA primer.

For dsRNA synthesis, genomic DNA was used as a template to amplify fragments of genes in Table 1, and the PCR products and the MEGA script RNAi Kit (Ambion Inc., Austin, Texas) were employed for dsRNA synthesis. Genomic DNA was extracted from *T. castaneum* adults and purified using the DNeasy Tissue Kit (QIAGEN, Valencia, CA). All the primers used for dsRNA synthesis and real time PCR are shown in Table 1. For annealing dsRNA, the reaction mixture was incubated at 75°C for 5 minutes and cooled to room temperature over a period of 60 minutes. After treatment with DNase, dsRNA was purified by phenol/chloroform extraction followed by ethanol precipitation. The dsRNA concentration was determined using a Nano Drop 2000 (Thermo Scientific, Pittsburgh, PA). The dsRNA was prepared using 808 bp PCR fragment of *E. coli malE* gene amplified from 28iMal vector (New England Biolabs, Ipswich, MA) was used as a control.

### dsRNA injection, topical application of JH III, and injection of bovine insulin

Newly hatched male adults (within 6 hours after emergence) were anesthetized with ether vapor for 4–5 minutes and lined on a glass slide covered with two-sided tape. The dsRNA was injected into the dorsal side of the first or second abdominal segment using an injection needle pulled from a glass capillary tube using a needle puller (Idaho Technology, Salt Lake City, UT). About 0.8–1 µg (0.1 µl) dsRNA was injected into each new male adult. The *malE* dsRNA was used as a control. The injected beetles were removed from the slide and reared in whole wheat flour at 30±1°C.

To restore the starvation survival by topical application of JH III or injection of bovine insulin, 0.5 µl of 10 mM JH III in acetone or acetone alone was topically applied to the males injected with *malE*, ILP2, or JHAMT dsRNA on day 3, day 5, and day 7. 0.2 µl 25 mM HEPES, pH 8.2, or 10 mg/ml bovine insulin solution in 25 mM HEPES (Sigma Aldrich, St. Louis, MO), was injected into *malE*, ILP2, or JHAMT RNAi males on day 5 PAE.

### Carbohydrate, lipid, and protein determination

Total amount of carbohydrates was determined using an Anthrone-based method [53]. A 1 µg/µl solution of glycogen was used as the standard, from which 0–200 µg calibration series were prepared. Three adults were placed in each tube and crushed with a homogenizer in 1 ml of Anthrone reagent. Standards and samples were heated at 92°C for 17 minutes. The samples were allowed to cool to room temperature and optical density (OD) was measured at 625 nm. The amount of total lipids was estimated using the vanillin reagent method [54]. A 1 µg/µl solution of commercial vegetable oil was used as a standard by preparing 0–400 µg calibration series. Three male adults were placed in each tube and crushed with a homogenizer in 500 µl mixture of chloroform–methanol. Samples were kept in a heating block to evaporate the chloroform–methanol. After evaporating the solvent, 200 µl of sulfuric acid was added, and samples were heated in a heating block at 99°C for 10 minutes. The samples were cooled to room temperature, and 800 µl of vanillin reagent was added to each tube and mixed well. Standards and samples were incubated for 30 minutes, and ODs of samples were read at 490 nm. Total protein levels were determined using the Bradford reagent (Sigma Aldrich, St. Louis, MO), and a series of dilutions of bovine serum albumin were used to prepare the standard curve.

### Trehalose and glucose determination in adults

To extract hemolymph from the beetles on days 4, 5, and 6 after injection of *malE*, ILP2, JHAMT, Met, TRET, or TPS dsRNA on day 0 newly emerged adults, the wings were removed and a few holes were poked into the body with forceps. The wings were placed in a microfuge tube containing 250 µl 0.25 M Na_2_CO_3_ buffer. The supernatant was collected after centrifugation at a full speed for 10 minutes. Trehalose is a non-reducing sugar resistant to 100°C. The hemolymph in the Na_2_CO_3_ buffer was incubated in a 95°C water bath for 2 hours to inactive all enzymes. 150 µl 1 M acetic acid and 600 µl 0.25 M Na-acetate (pH 5.2) were added, and the solution was centrifuged (10 minutes, 12,000 rpm, 24°C). One hundred microliters of supernatant were incubated overnight at 37°C with 1 µl porcine kidney trehalase (Sigma Aldrich, St. Louis, MO) to convert trehalose into glucose. Thirty microliters of this solution were added to 100 microliters of a glucose reagent solution (Sigma Aldrich, St. Louis, MO) and incubated 20 minutes at 37°C. Glucose concentration was quantified at 340 nm with a spectrophotometer. The trehalose dihydrate (Sigma Aldrich, St. Louis, MO) was used as a control and also used to prepare reference curves.

### The survival assay

Newly emerged beetles were injected with dsRNA and reared without diet in the 96-well plate individually at 30°C incubator and checked at 5:00 pm every day. Male adults were used in all the experiments.

### Statistical analysis

All the data were analyzed using the SPSS 13.0. The Kaplan-Meier program was used to analyze the survival time and the Log rank analysis was performed to compare the effect of JH III and insulin treatment. To compare nutrient levels, mRNA levels or the ratio between glucose and trehalose, the one-way ANOVA was used, for all the data analysis, the *P*-*value* for statistical significance is defined as *P*<*0.05*.

## Supporting Information

Figure S1 The knockdown efficiency for all the genes tested in this study. The bars show percentage of gene expression compared to that in beetles injected with *malE* dsRNA set as 100. The total RNA was isolated from the whole body on day 4 and used to quantify relative mRNA levels for each gene by qRT-PCR using RP49 as an internal control.
(TIF)

Figure S2 Alignment of *T. castaneum* TRET, trehalase, and TPS amino acid sequences with the amino acid sequences of closely related homologs. A. TcTRET amino acid sequence is aligned with AgTRET (*Anopheles gambiae*, AGAP005563-PA), BmTRET (*Bombyx mori*, NP 001108344.1), DmTRET (*Drosophila melanoganster*, Q8MKK4.1) and LmTRET (*Locusta migratoria*, AAT72921.1) B. Tctrehalase amino acid sequence is aligned with Agtrehalase (*Anopheles gambiae*, EAA00681.4), Bmtrehalase (*Bombyx mori*, BAE45249), Cqtrehalase (*Culex quinquefasciatus*, EDS26356.1), and Dmtrehalase (*Drosophila melanoganster*, ABH06691.1) C. TcTPS amino acid sequence is aligned with AaTPS (*Aedes aegypti*, EAT41968.1), AgTPS (*Anopheles gambiae*, EAA12459.4), CqTPS (*Culex quinquefasciatus*, EDS32889.1), DmTPS (*Drosophila melanoganster*, AAD38628.1).
(TIF)

Figure S3 JH regulates ILP2 expression in starved beetles. A. The relative mRNA levels of ILP2 in day 5 starved males injected with either JHAMT, Met, or *malE* dsRNA. Total RNA was isolated from the whole body at 6 hours after treatment on day 4. The total RNA was converted to cDNA, and the relative ILP2 mRNA levels were determined by qRT-PCR using RP49 as an internal control. The data shown are the Mean+S.D. (n = 3). B. The relative mRNA levels of ILP2 in day 5 starved males injected with malE or ILP2 dsRNA soon after adult emergence and topically applied with either acetone or JH III. Total RNA was isolated at 6 hours after application from the whole body and used to measure relative ILP2 mRNA levels by qRT-PCR using RP49 as a control. The data shown are the Mean+S.D. (n = 3). Asterisks show treatments that are significantly different from the control (*P*<0.05) in one-way ANOVA.
(TIF)

Figure S4 JH and insulin regulate starvation survival. Percentages of beetles survived after knockdown of JHAMT, Met, or ILP2 are shown. The dsRNA of *malE*, JHAMT, or Met was injected into day 4 males feeding on normal diet (40 males for each dsRNA). The beetles were starved after dsRNA injections, and survival was recorded from day 9 to day 14.
(TIF)

Figure S5 The relative mRNA levels of JHAMT and Kr-h1. A. The relative mRNA levels of JHAMT in starved and fed male beetles. Total RNA was isolated from starved and fed beetles collected on days 4, 5 and 6 PAE and used to measure relative JHAMT mRNA levels by qRT-PCR using RP49 as a control. The data shown are the Mean+S.D. (n = 3). Asterisks show treatments that are significantly different from the control (*P*<0.05) in one-way ANOVA. B. Same as in Figure S5 A except Kr-h1 mRNA levels were quantified.
(TIF)
