# Supplementary figures and images for: Juvenile Hormone and Insulin Regulate Trehalose Homeostasis in the Red Flour Beetle, Tribolium castaneum
Source: PLoS Genet. 2013 Jun 6;9(6):e1003535. doi: 10.1371/journal.pgen.1003535 (PMC3675034; doi:10.1371/journal.pgen.1003535)

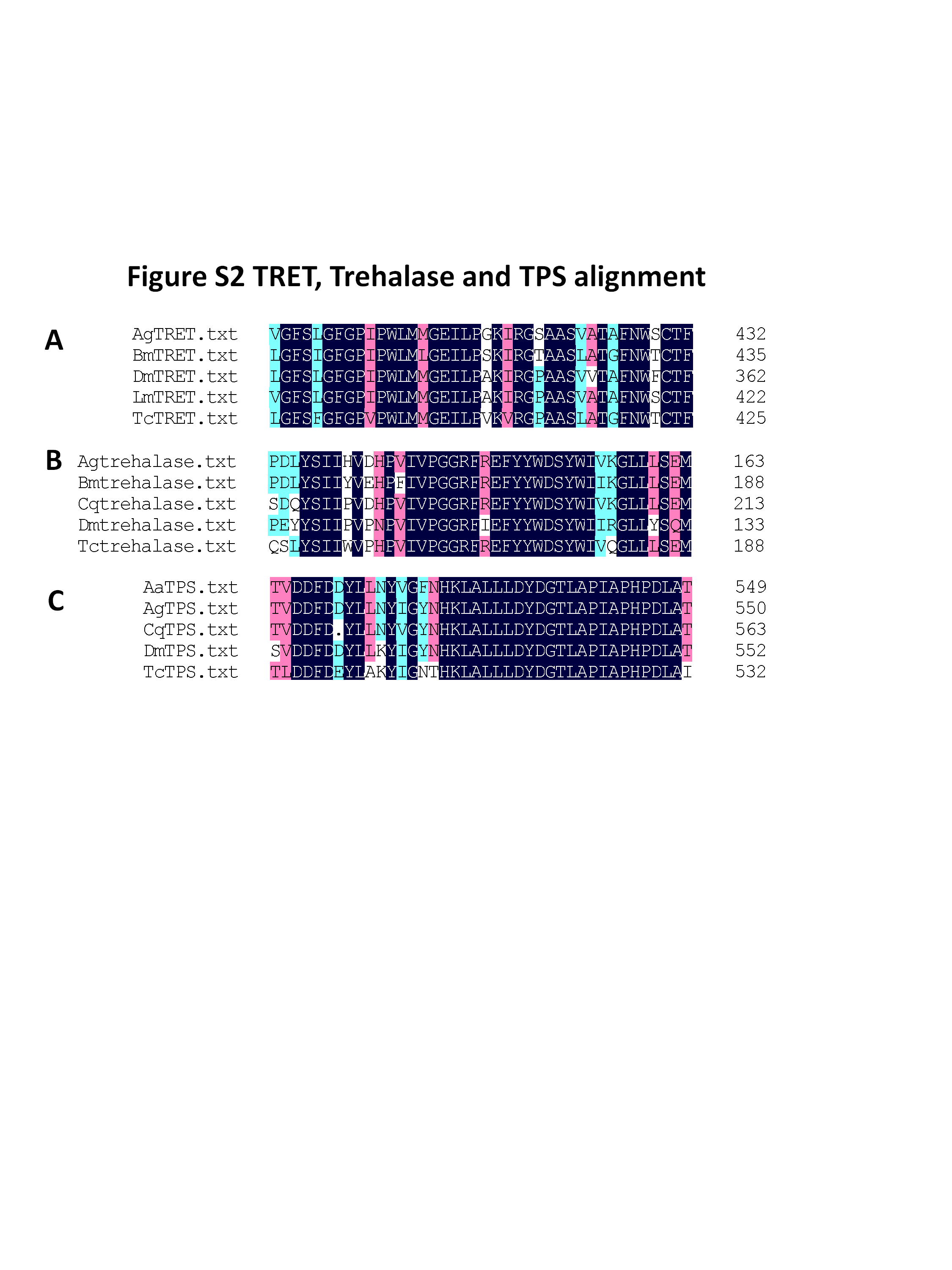

Supplement: Figure S2 — Alignment of T. castaneum TRET, trehalase, and TPS amino acid sequences with the amino acid sequences of closely related homologs. A. TcTRET amino acid sequence is aligned with AgTRET (Anopheles gambiae, AGAP005563-PA), BmTRET (Bombyx mori, NP 001108344.1), DmTRET (Drosophila melanoganster, Q8MKK4.1) and LmTRET (Locusta migratoria, AAT72921.1) B. Tctrehalase amino acid sequence is aligned with Agtrehalase (Anopheles gambiae, EAA00681.4), Bmtrehalase (Bombyx mori, BAE45249), Cqtrehalase (Culex quinquefasciatus, EDS26356.1), and Dmtrehalase (Drosophila melanoganster, ABH06691.1) C. TcTPS amino acid sequence is aligned with AaTPS (Aedes aegypti, EAT41968.1), AgTPS (Anopheles gambiae, EAA12459.4), CqTPS (Culex quinquefasciatus, EDS32889.1), DmTPS (Drosophila melanoganster, AAD38628.1). (TIF) [file pgen.1003535.s002.tif]

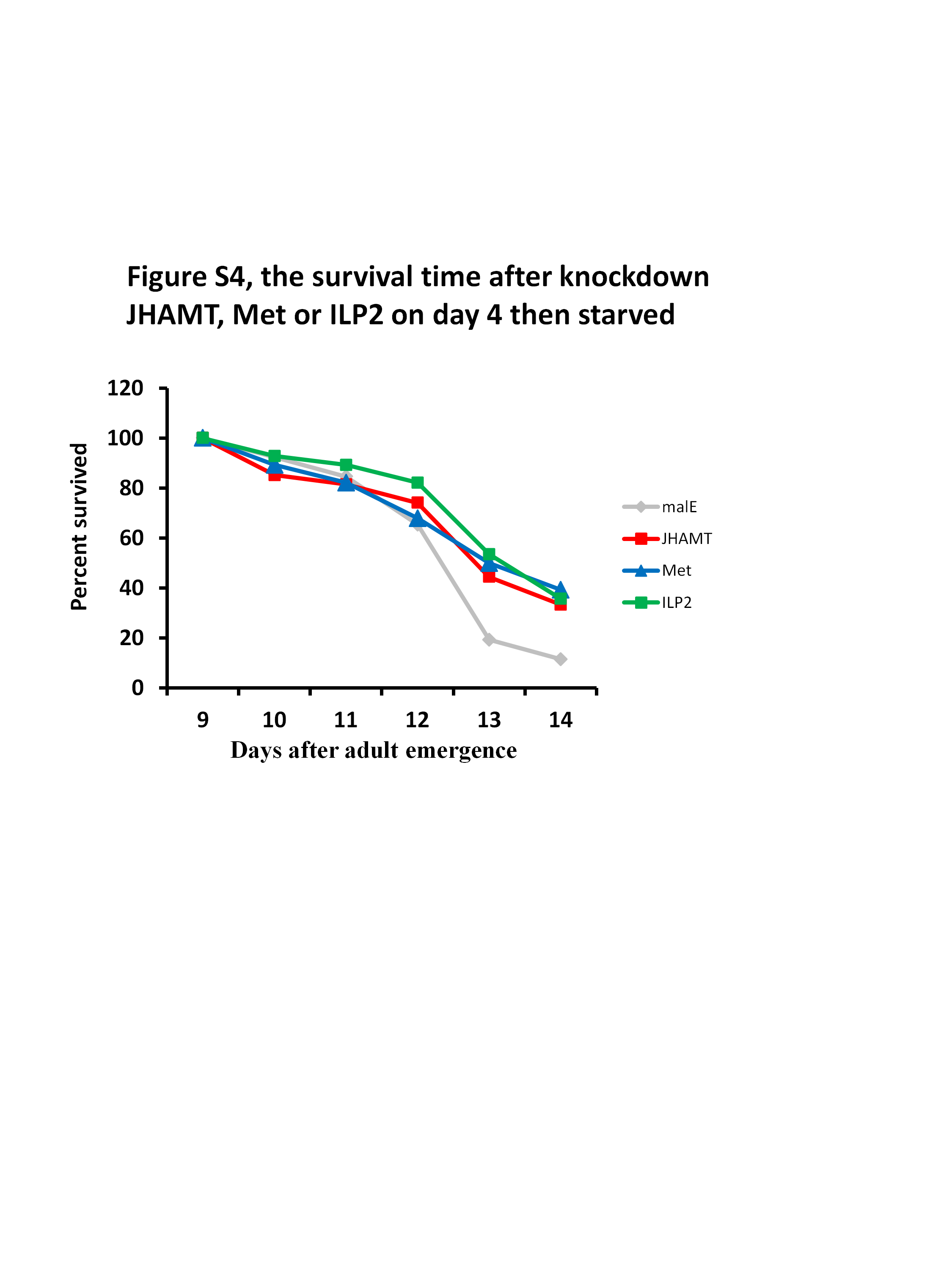

Supplement: Figure S4 — JH and insulin regulate starvation survival. Percentages of beetles survived after knockdown of JHAMT, Met, or ILP2 are shown. The dsRNA of malE, JHAMT, or Met was injected into day 4 males feeding on normal diet (40 males for each dsRNA). The beetles were starved after dsRNA injections, and survival was recorded from day 9 to day 14. (TIF) [file pgen.1003535.s004.tif]
